# A New Bottle Design Decreases Hypoxemic Episodes during Feeding in Preterm Infants

**DOI:** 10.1155/2012/531608

**Published:** 2012-06-20

**Authors:** Alejandro Jenik, Carlos Fustiñana, Maritza Marquez, David Mage, Gloria Fernandez, Gonzalo Mariani

**Affiliations:** ^1^Departamento de Neonatología, Hospital Italiano de San Justo Agustín Rocca, Avenue Presidente Perón 2231 San Justo, La Matanza, Provincia de Buenos Aires (B1754AZK), Argentina; ^2^U.S. Environmental Protection Agency, National Exposure Research Laboratory, Research Triangle Park, NC, USA

## Abstract

Oxygen saturation is lower during bottle feeding than during breastfeeding in preterm infants. Our objective was to compare two different bottle systems in healthy preterm infants before discharge in terms of SpO_2_ and oral feeding efficiency (rate of milk intake). Infants without supplement oxygen needs were evaluated twice on the same day during two consecutive feeds, by the same nurse. Infants served as their own controls for comparison of two systems of bottles, the order of which was randomized. The new bottle's nipple design mimics mom's breast in shape and feel, and the bottle vents to air when the child sucks on the nipple. The other system was the hospital's standard plastic bottle with silicone nipple. The rate of milk intake was calculated as the total volume transferred minus volume lost divided by time of feeding, mL/min. Thirty-four infants (BW: 1, 163 ± 479.1 g) were studied at 35.4 ± 1.3 weeks after-conception. SpO_2_ was significantly higher in infants fed with the new bottle design. Milk intake rate was significantly higher with the new bottle than with the standard bottle design. The new bottle design improves oral feeding performance in preterm infants near to discharge when compared to that of a standard bottle.

## 1. Introduction

Preterm infants who receive expressed breast milk rather than formula have fewer infections and necrotizing enterocolitis, as well as other better developments [1]. However, mothers of vulnerable infants, such as neonates, encounter a variety of unique breastfeeding barriers and challenges that may result in a decreased rate of breastfeeding. In these cases, the next “best” alternative is bottle feeding with expressed breast milk. However, the characteristics of commercial bottle systems vary widely in terms of flow rates achieved, and some may require well-developed sucking capabilities—which may be problematic in premature babies. The main purpose of this research is to provide much needed information on how a premature baby tackles being fed expressed milk from different bottle systems in terms of oxygen saturation (SpO_2_) and oral feeding efficiency.

Improving oral feeding skills accelerates attainment of independent oral feeding, shortens hospitalization, and reduces medical costs, allowing earlier family reunification and development of more appropriate mother-infant interaction and bonding. Infant feeding is a complex process, requiring the precise coordination of sucking, and pharyngeal swallowing and breathing which are mutually exclusive. Therefore, the pharynx must be continually and rapidly reconfigured so that baby can successfully eat and breathe almost simultaneously. Unlike artificial nipples, the human breast transforms to fit the shape, size, and positioning of the infant's mouth. In addition, the magnitude and consistency of milk flow from a milk bottle are quite different from that of the breast, and milk bottles may generate excessive internal negative pressure, which may collapse the nipple and provide resistance to infant sucking. 

Because oxygen saturation is higher during breastfeeding than bottle feeding [2, 3] there may be a mechanistic basis for the advantage from differences in tongue posture and less disruption of breathing. By extension, there may be differences among bottle feeding systems, some of which may promote more natural postures and breathing patterns than others. Breastfeeding involves tongue and jaw movement to “strip milk” from the breast's natural milk ducts, while bottle-feeding involves sucking. Breastfed babies do not learn to release, gasp, and reattach while feeding because the breast does not build up an internal vacuum, nor does it collapse. This is one of the reasons why breastfed babies have such a hard time transitioning to a regular baby bottle. When babies nurse from regular reusable bottles, vacuum builds up within until the nipple collapses on itself. Babies who are bottle-fed learn to release the nipple and gasp air through their nose and mouth and then resume sucking on the bottle. 

Our hypothesis is that use of a new bottle system (developed by MAM Babyantikel GesmhH) when compared to our hospital's standard firm-silicone bottle (without valve and vents and with traditional bottle nipple shape feeling unlike mother's breast) will reduce stress of oxygen desaturation and improve feeding outcome. We compare and evaluate them to judge which comes closest to “gold-standard” breast feeding. The new MAM bottle features the ULTIVENT valve and nipple, which interact with each other to replicate breastfeeding.

## 2. Methods

### 2.1. Subjects

Infants were recruited from the Neonatal Intensive Care Unit (NICU) at Hospital Italiano of San Justo Agustin Rocca, Buenos Aires, Argentina. A total of 34 clinically stable preterm neonates, postconceptional age (PCA) >34 weeks, were chosen as eligible for the study. They were near the time of discharge from the NICU, were not receiving oxygen, and were exclusively oral-feeding 100% of their daily milk intake. These preterm infants were evaluated and enrolled after obtaining written informed parental consent if they were (1) born with >27 weeks gestational age (GA) as determined by obstetrical ultrasound and clinical exam; (2) without medical problems that might influence feeding; (3) of appropriate size for GA; (4) without known congenital anomalies; (5) without chronic medical conditions including bronchopulmonary dysplasia, intraventricular hemorrhage grade III or IV, periventricular leukomalacia, or necrotizing enterocolitis; (6) not exclusively breastfeed; (7) free of any observed episodes of apnea, bradycardia, or significant oxygen desaturation prior to evaluation.

### 2.2. Physical Properties of Baby Bottles

#### 2.2.1. Standard Bottle (Figure 1)

The standard bottle used in this study was straight and cylindrical in shape, without valve and vents and with traditional bottle nipple shape feeling unlike mother's breast. Vacuum builds up within the bottle as an infant withdraws milk. As feed progresses, this negative pressure/force increases, opposing the suction exerted by the infant. This situation leads to difficulty in generating suction and/or results in a decrease in the rate of milk flow. 

#### 2.2.2. New Bottle Design (Figure 2(a)) and New Nipple Design (Figure 2(b))

The shape, design, placement, and construction of the valve within the new bottle design allow for a continuous and ultrasensitive response, preventing a buildup of internal vacuum. The center of the valve opens and closes to release vacuum from the bottle in a continuous process during nursing. This continuous opening and closing process is what allows the nipple to react like the breast. It allows the nipple to deliver milk in an uninterrupted process just like the breast during breastfeeding. 

For the same sucking effort the new bottle will give a higher flow rate because the atmospheric pressure in the bottle above the milk will help push the milk whereas a partial vacuum above the milk will retard the flow to baby.

**Figure 2:**
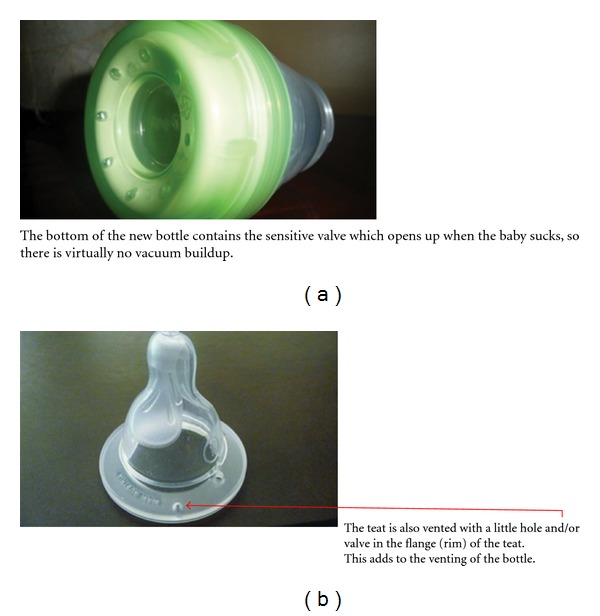
(a) New Bottle design. (b) The new bottle's nipple.

The newly designed nipple (Figure 2(b)) mimics mom's breast in shape and feel. The breast-shaped nipple contains internal Rib structures to encourage “stripping” action and eliminate nipple collapse. The silky textured area replicates the silky soft skin of the mother's breast. The broad bulge of the teat allows sufficient space for the lips to latch on, just like the breast, so the baby can uninterruptedly feed without swallowing air.

**Figure 3:**
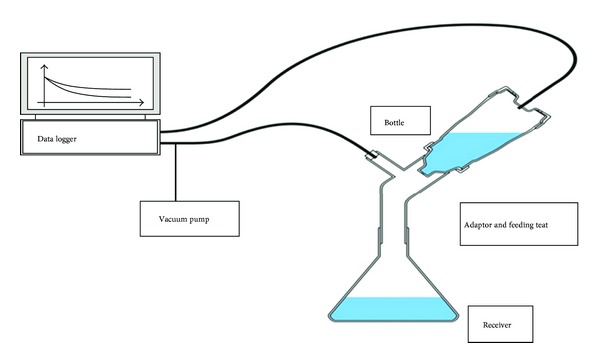
Schematic view of the bottle testing apparatus.

The new bottle was tested by using a sucking device (Figure 3). 

The main features of the apparatus are as follows.

 The sucking force generated by the Vacuum Pump may be accurately varied to within 1 mmHg. It is connected to the Receiver and hence allows suction to be applied to the Adaptor and Feeding Teat.
 The Feeding Teat is held in place in the Receiver with an Adaptor designed for the shape of the particular feeding teat being tested.
 Via a hole drilled in the bottom of the standard bottle or side of the new Bottle, the internal negative pressure (or vacuum) in the bottle can be continuously recorded in a Data Logger which is also designed to record the sucking pressure exerted by the vacuum pump.
  Tap water was used as a simulant for milk throughout the tests.
 The red trace in the charts below is the “Sucking pressure” exerted by the vacuum pump on the bottle.
 The blue trace (called by the data logger “Measuring pressure”) is a measurement of the internal negative pressure in the air space in the bottle.

Two representative tests are presented as examples.

**Figure 4:**
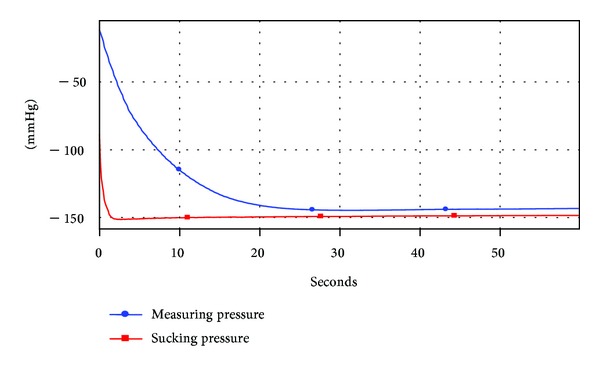
Performance of conventional unventilated bottle with an unvented teat.

**Figure 5:**
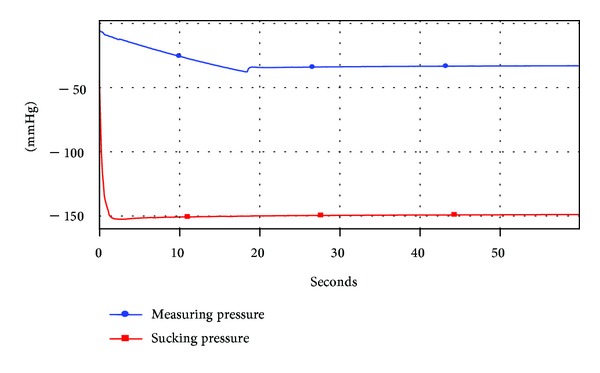
Performance of new bottle design with a vented teat.

 This uses a sucking pressure of up to 150 mm. The internal pressure of the bottle quickly falls (~110 mmHg after 10 seconds) and reaches a minimum after 27 seconds (see Figure 4).
 The fall of internal pressure is much slower (circa 25 mmHg after 10 seconds).
 The bottle ventilation initiates after 18 seconds at −38 mmHg and maintains a slightly reducing negative internal pressure (−33 ± 1 mmHg) for the remainder of the trial (see Figure 5). What Is the Difference with Feeding Function between the New Bottle Design and the Standard Bottle in terms of the Function of Mother's Breast?

The physiological pressure in the milk duct forcing out the milk from the nipple remains the same high value while baby is sucking. This is similar to the new bottle's valves which keep atmospheric pressure above the milk the same. So the new nipple correctly mimics the mom's constant breast pressure difference.

## 3. Study Design 

Infants were evaluated twice on the same day during two consecutive feedings by the same research nurse, who was blinded to the oxygen saturation (SpO_2_) recording. They were studied five minutes before and throughout a bottle feeding at 3 h intervals using the two different bottles: a new design bottle/nipple or a conventional bottle/nipple. The sequence in which these bottles were given was determined using a random allocation procedure. Infants served as their own controls for comparison of bottle feedings and all feedings were performed with their mother's expressed milk. For cases in which the amount of mother's milk was insufficient, the volume was supplemented with a milk formula adequate for preterm infants. To ensure that we did not interfere with breastfeeding, assessments were made when mothers were absent. 

## 4. Oral Feeding Outcomes

The principal aim of our study was to compare the effects of bottle-feeding using a new bottle design (MAM Ultivent bottle) versus the hospital's standard bottle on the occurrence, severity, and pattern of oxygen desaturation in healthy preterm babies near the time of discharge from the NICU. The secondary outcome was a comparison of the oral feeding efficiency during the use of the two systems of bottle feeding. Oral feeding efficiency was measured by the rate of milk intake and percent milk leakage or loss. 

## 5. Data Collection 

SpO_2_ was recorded continuously with the Nellcor pulse oximetry channel of the EdenTrace II Plus AMS (Mallinckrodt, St. Louis, MO). Data were stored and recovered for analysis. The oximeter probe was placed on the infants' foot in a position that provided an optimal signal. Clinically significant desaturation events were defined as any decrease in SpO_2_ below 90% for 1 s or more. In addition, instantaneous severe drops in SpO_2_ were considered artifacts. We considered the following desaturation variables: (1) the percentage of feeding time SpO_2_ < 90%; (2) percentage of feeding time SpO_2_ 90%–94%; (3) the number of desaturation events per infant feeding; (4) the time with SpO_2_ < 90% and (5) mean SpO_2_ during feeding. The average rate of milk intake (mL/min) was considered as the total volume transferred minus volume lost during feeding divided by the feeding time which was defined as the time from the first nipple in mouth through the final nipple out. The duration of feeding included the “out” times during which the infant needed to burp, cough, or rest. These decisions were left to the discretion of the research nurse who assessed infant's need. The percent milk leakage or loss was measured by weighing a bib before and after each feeding (an increase of 1 g = 1 mL of loss) over the total volume of milk removed from the bottle.

## 6. Sample Size

Taking into account that NICU infants currently spend 20% of their bottle feeding time with SpO_2_ levels <90% [4], our expectation is that infants fed with the new MAM Ultivent system will increase the oxygen saturation sufficiently so the infants will spend 10% or less of their time with SpO_2_ levels <90%. A sufficient sample size of 34 preterm infants was calculated with an alpha level of 0.05, using a type 1 error of 0.05 and a power of 0.80.

## 7. Statistical Analysis

Data were analyzed with the Epi-Info 6.02, Stata 5.0. Descriptive statistics were calculated for infant and maternal characteristics (baseline SpO_2_,body weight (BW), gestational age (GA), PostConceptionalAge (PCA)) and maternal parity, feeding length, and desaturation variables. The Student's *t*-test was used to analyze data showing a normal distribution and the nonparametric Wilcoxon test was used for variables that did not show a normal distribution. In this last case results are presented as medians and interquartile ranges.

## 8. Ethical Considerations

The study was approved by the Hospital Institutional Review Board for Protection of Human Subjects, and a signed written informed parental consent was obtained before each study pair of feedings.

## 9. Results

Thirty-four preterm infants were studied. Infants and maternal characteristics are summarized in Table 1. Infants did not differ in PCA at the time of the study (range 35-36 weeks) and were near discharge (mean 2.4 days). Baseline SpO_2_ was within a clinically acceptable range.

### 9.1. Desaturations Events

Descriptive data of the desaturation events are provided in Table 2. Preterm infants who are normally oxygenated in room air have significant desaturation during bottle feeding. However, taking into account all the desaturation variables, the SpO_2_ during feeding was significantly higher in infants fed with the new bottle design compared with the standard bottle. Of all the minutes of the feeding, the initial minute of the feeding had the highest number of desaturations both with the new design bottle as with the standard bottle. There was no significant effect of feeding bottle on the frequency of apnea or bradycardia.

### 9.2. Oral Feeding Outcomes

**Figure 6:**
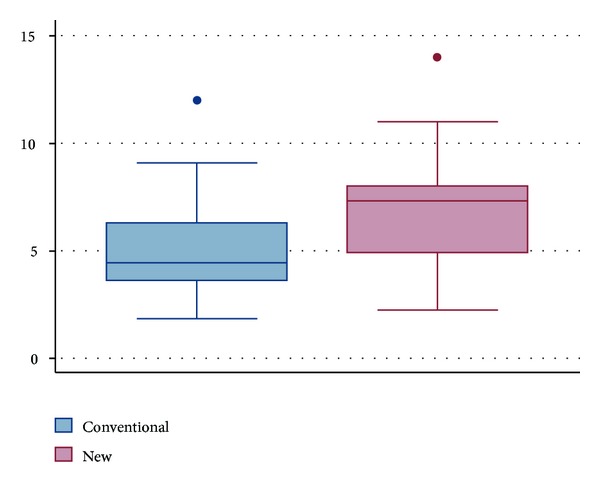
Rate of milk transfer (mL/min).

Rate of milk transfer was statistically different between the two bottle nipples. Rate of milk transfer (Figure 6) was significantly less with the standard bottle than with the new bottle design (5.0 ± 2.2 Versus 6.9 ±2.6 ml/min, resp., *P* < 0.0001). 

**Figure 7:**
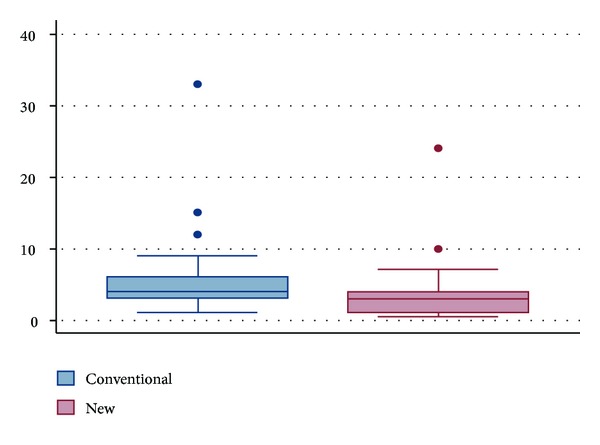
Percent milk loss.

Percent milk loss (Figure 7) decreased with the new bottle design when we compared it to the standard bottle (3.5 ± 4.3 Versus. 5.4 ± 5.6%, resp., *P* < 0.0001). 

## 10. Discussion

 Preterm infants who are normally oxygenated in room air may have significant desaturation during bottle feeding [5]. With the increased survival of preterm infants, awareness of oral feeding difficulties in this population is growing. A temporal association between feeding and episodes of cyanosis in preterm infants was first noted more than 90 years ago [6]. In the present study, we confirm our hypothesis that the use of the new design bottle when compared to that of standard bottle improves oral feeding performance in infants born with 27 week gestation or more in terms of oxygen saturation and oral feeding efficiency, that is, greater rate of milk intake and less percent milk leakage/loss. 

Oxygen desaturation during bottle feeding is impacted by the feeding condition and the health of the infant. Previous studies have consistently demonstrated that breastfed babies have higher oxygen saturation than bottlefed babies. A frequently cited explanation for this difference is that bottle-feeding may promote a higher rate of swallowing and, in turn, more frequent interruptions of breathing. Indeed, studies have shown that there is less ventilatory disruption during breast-feeding compared with bottle feeding, which may result in higher oxygen saturation [7]. 

 Infants with lower oxygen saturation tend to have shorter sucking bursts, potentially signifying less energy available for sucking. They also tend to organize restorative breathing between sucking bursts poorly, engaging instead, in shorter intervals between sucking bursts [8]. 

We have demonstrated that VLBW infants continue to have frequent oxygen desaturation events during bottle feeding near the time of discharge from the NICU. Thoyre and Carlon have demonstrated in an elegant paper that preterm infants at the postconceptional age of 36.5 ± 1.6 weeks spent on average 20% of their bottle feeding time with oxygen levels below 90%, near the time of discharge [4]. These values are significantly lower than the normal range of oxygen saturation for preterm infants during nonfeeding periods. The preterm infants included in our study showed higher oxygen saturation during the feeding period compared with Thoyre and Carlson's study [4]. This fact is explained because they included 30% of their patients with supplemental oxygen requirements. Our study population has included only healthy preterm infants prior to discharge from the hospital. We speculate that preterm infants showed less desaturation events during the feeding process with the new design bottle because the nipple resembles the mother breast in terms of shape and texture of the silicone, that is, much softer and silkier in comparison to the standard nipple. The shape, design, placement, and construction of the valve within the new design bottle (a large vent with a large center hole) prevent a high buildup of internal vacuum. Adequate oxygenation enables infants to maintain behavioral organization [9]. Lower oxygen saturation during feeding impacts the infant's ability to organize and maintain oral feeding skills. The low oxygen saturation observed in the infants when they were fed with the standard bottle explains the poorer feeding outcomes in regards to the preterm infants fed with the new design bottle. 

In summary, although breastfeeding is clearly best for infants, it may not always be possible. Our results suggest that the overall feeding pattern and oxygenation of the new model design bottle are closer to the physiologic norm than the standard bottle. The new design bottle would be very useful for some preterm infants, particularly those with bronchopulmonary dysplasia that exhibit significant desaturation during and immediately after bottle feeding [10]. The results of this study have several practical implications for evaluating issues of great concern to women who breast-feed but who may choose to intersperse some bottle and artificial nipple feeding with breast milk while they are at work.

## Figures and Tables

**Figure 1:**
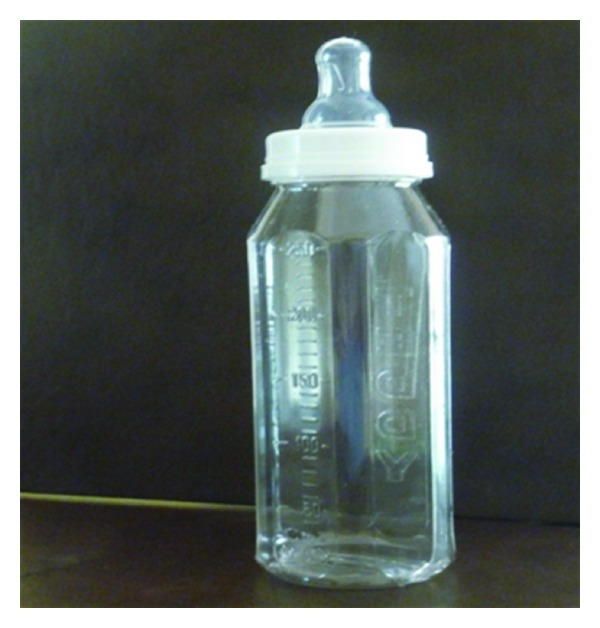
Standard bottle.

**Table 1:** Characteristics of the sample (*N* = 34): 14 males, 20 females.

Variables	Mean	S.D.
Infants		
BW (g)	1163.4	479.1
GA (weeks)	31.1	3.1
PCA (weeks)	35.4	1.3
Days on oxygen (prior to study)	6.3	10.4
Days before discharge	2.4	1.61
SpO_2_ during baseline period (%)	96.8	1.90
Mothers		
Age (years)	29.06	4.72
Education (years)	13.93	3.41
Parity	2	1.8

**Table 2:** Oxygen desaturation events, expressed as median and (interquartile range).

	Standard bottle	New bottle design	*P* ^ ∗^
Percentage of feeding time SpO_2_ <90%	8% (3–13)	5% (2–11)	<0.004
Percentage of feeding time SpO_2_ 90% −94%	13% (6–21)	8% (2–18)	<0.0007
Number of desaturation events per infant	10 (1–19)	4 (1–8)	<0.001
Time with SpO_2_ <90% (s)	46 s (8.3–150 )	30 s (6–96)	<0.001
Mean SpO_2_ during feeding	94 (91–96)	96 (93–98)	<0.0008

^∗^Wilcoxon signed-0 rank test.
